# Optimization and Predictive Modeling of SiC Wafer Dicing Using a Thin Diamond Grinding Wheel via RSM and NSGA-II

**DOI:** 10.3390/mi17060686

**Published:** 2026-06-01

**Authors:** Jian Liu, Meiling Du, Jinzhong Wu, Sheng Gong, Penggen Ouyang, Shuai Huang, Fengjun Chen

**Affiliations:** 1 School of Robot Engineering, Wenzhou University of Technology, Wenzhou 325000, China; 20220048@wzut.edu.cn (J.L.); dumeiling@stu.wzu.edu.cn (M.D.); abccfj@126.com (F.C.); 2 School of Intelligent Engineering, Shaoxing University, Shaoxing 312000, China; 3 Hefei Accuracy Intelligent Equipment Co., Ltd., Hefei 230094, China; gshryl@126.com; 4 Zhejiang Jingrui Electronic Materials Co., Ltd., Shaoxing 312000, China; ouyangpenggen@jsjd.cc; 5 College of Mechanical and Vehicle Engineering, Hunan University, Changsha 410082, China; huangshuai9228@126.com

**Keywords:** dicing, SiC wafer, diamond grinding wheel, process parameters, response surface analysis

## Abstract

To investigate how the process parameters of ultra-thin diamond grinding wheel dicing affect the dicing quality of silicon carbide (SiC) wafers, single-factor experiments were designed. This study examined the influence of key process parameters, including spindle speed, feed rate, and first dicing depth, on the maximum chip width on the front side W_1_ and the maximum chip width on the back side W_2_, thereby determining their optimal parameter ranges. Subsequently, a quadratic polynomial prediction model was established using response surface analysis to analyze the interactive effects among the grinding wheel dicing process parameters. Finally, the prediction model was optimized using the genetic algorithm NSGA-II, and the optimal parameter combination for the two response variables was determined: a spindle speed of 31,960 r/min, a feed rate of 2.0019 mm/s, and a first dicing depth of 197.51 μm, yielding an average W_1_ of 4.8852 μm and W_2_ of 18.5360 μm. The relative errors between the predicted and average experimental values are 2.83% for W_1_ and 4.43% for W_2_. Both errors are below 5%, confirming the validity of the model. Therefore, the model serves as a practical reference for planning subsequent dicing processes using ultra-thin diamond grinding wheels.

## 1. Introduction

Semiconductor materials offer significant development potential in China’s integrated circuit, optoelectronics, aerospace, and defense sectors. Among these, the third-generation semiconductor material silicon carbide (SiC) is widely used in high-temperature, high-frequency, radiation-resistant, and high-power devices owing to its excellent physical properties. Generally, higher SiC content and purity lead to enhanced physical properties. However, the extremely high brittleness of SiC makes it susceptible to edge chipping during dicing. Diamond wheel dicing is currently one of the mainstream dicing technologies for SiC wafers on the market, and the quality of the dicing directly affects product yield and cost [1,2,3,4].

Numerous factors influence the dicing quality of diamond grinding wheels, primarily including three key process parameters: spindle speed, feed rate, and dicing depth, as well as blade parameters. Since these parameters interact with one another, the appropriateness of their settings directly affects the width of the chipped edge during dicing [5,6]. Araujo et al. [7] conducted high-precision machining of high-alumina substrates with a grinding wheel dicer. By varying dicing depth, feed rate, and spindle speed, they analyzed blade wear and dicing quality, and subsequently investigated the material removal mechanism. The experimental results demonstrated that operating at the high spindle speed of 30,000 r/min enhances blade rigidity, leading to stable dicing and good edge quality. Li et al. [8] investigated the high-speed grinding of silicon wafers using diamond-coated dicing blades. Through analysis of the grinding mechanism, the effects of spindle speed and feed rate on dicing quality were evaluated, and the cross-sectional shape, edge length, diamond particle concentration, and blade thickness were subsequently optimized. The experimental results demonstrated that the optimal diamond particle wear rate, dicing quality, and machining efficiency were obtained at a spindle speed of 50,000 r/min, feed rate of 60~80 mm/s, and dicing depth of 30, 50, or 75 μm. Therefore, understanding how various parameters affect dicing quality is essential for determining a reasonable set of parameters using a specific optimization method. This approach not only provides theoretical guidance for future researchers but also offers practical and reliable references for engineering applications.

Yin et al. [9] proposed a layered dicing process for silicon wafers and investigated, relative to the traditional single-pass dicing process, the effects of key process parameters, namely spindle speed, feed rate, and dicing depth, on the performance of this layered approach. The objective was to optimize these parameters to reduce the width of the chipped edges. Yan et al. [10] designed a focused ultrasound transducer that utilizes the flow field and cavitation effects of sound waves. By adjusting the linear velocity and normal load to induce high-frequency vibration in a diamond wire, the cutting force during the SiC cutting process was reduced, thereby decreasing the maximum chipping width by 56.6%. Su et al. [11] used analysis of variance to identify key optimization parameters when studying the chipping width of silicon wafer dicing grooves, thereby improving the chipping resistance of the grooves. Using orthogonal experiments and genetic algorithms, Shi et al. [12] optimized dicing process parameters, achieving an optimized average maximum dicing width of 38.54 μm, which represents an 8.23% improvement over conventional scribing. Ma et al. [13] employed orthogonal experiments to investigate the effects of spindle speed, feed rate, dicing depth, and tool diameter on dicing quality. Using range analysis, analysis of variance (ANOVA), and factor–index trend plots, the optimal parameters were determined based on how each parameter influenced dicing width. Sun et al. [14] selected a range of process parameters based on avoiding the natural frequencies of various components. With the root mean square value of spindle vibration as the evaluation metric, a regression equation was established through orthogonal experimental design and iteratively optimized via a genetic algorithm to determine the optimal process parameters. Kurniawan et al. [15] proposed a novel ultrasonic dicing blade to address edge chipping and flaking issues associated with conventional silicon wafer dicing. Although the proposed blade outperformed conventional counterparts, no optimization study of the dicing process parameters was conducted. Chang et al. [16] employed a bidirectional long short-term memory (BLSTM) network for chipping prediction on a dicing machine. The model was trained to address robustness issues across machines, achieving high-precision chipping prediction and thereby optimizing dicing parameters to reduce losses. Currently, however, most research has focused on the relationships between dicing process parameters and quality metrics for silicon wafers, with few studies specifically optimizing SiC or its related parameters.

Multi-objective optimization algorithms serve as methods for comprehensively considering the impacts of various parameters on the overall objective, and they are often suitable for specific combinatorial optimization problems. Chen et al. [17] developed a material removal depth model for end-face polishing based on the Preston equation and derived a material removal rate model. A quadratic polynomial model for predicting surface roughness was constructed through orthogonal experiments, with its reliability validated by analysis of variance. Finally, the multi-objective particle swarm optimization algorithm was employed to obtain a set of Pareto-optimal solutions and determine the optimal combination of process parameters. Mukras et al. [18] applied a multi-objective genetic algorithm to resolve product quality defects arising from various injection molding process parameters, with defect minimization as the primary goal. Experimental results yielded an error margin of roughly 7%. Kahhal et al. [19] combined response surface methodology with multi-objective particle swarm optimization for the multi-objective optimization of mechanical properties in aluminum alloy friction stir welding. Experimental results demonstrate that the modeling and optimization of this problem not only improved tool pin accuracy but also reduced testing costs. Zhang et al. [20] addressed the local optima issue encountered by the non-dominated sorting genetic algorithm II (NSGA-II) during multi-objective optimization and proposed an improved NSGA-II algorithm to handle this problem. Through the optimal combination of key process parameters, this approach effectively improved workpiece surface roughness and material removal rate, further demonstrating the superiority of the improved NSGA-II algorithm in machining process optimization. Patil et al. [21] combined Taguchi experimental design with the non-dominant sorting genetic algorithm (NSGA-II) for simultaneous optimization of surface roughness, power consumption, and the grinding ratio, thus determining the optimal parameter combination. Experimental results yielded the best surface roughness of 0.2795 μm, grinding power of 1.696 kW, and grinding ratio of 11.1621, providing an effective solution. The combination of polynomial equations with genetic algorithms not only improves optimization performance but also prevents the optimization process from being trapped in local optima. During the optimization of process parameter combinations for SiC dicing, combining multivariate equations derived from the response surface method with traditional genetic algorithms readily causes the optimization process to fall into local optima. Hence, this paper employs an NSGA-II multi-objective optimization algorithm for the determination of optimal process parameter combinations.

SiC is classified as a typical hard and brittle material. The complex physical properties of this material often lead to various defects, including chipping and material delamination, during the dicing process when improper process parameters are used. With the maximum chip width on the front side (W_1_) and the maximum chip width on the back side (W_2_) as evaluation criteria, single-factor experiments were employed to investigate the influence of SiC wafer dicing process parameters on chip width, thereby determining optimal ranges for spindle speed, feed rate, and dicing depth. A Box–Behnken response surface design was conducted. Analysis of variance (ANOVA) on W_1_ and W_2_ established a quadratic regression model that relates the response variables to the independent variables. The interaction response surfaces were subsequently analyzed to reveal the interrelationships between the process parameters and performance metrics.

The quadratic regression model was optimized using the NSGA-II algorithm, yielding the optimal parameter combination. Experimental validation assessed prediction errors.

## 2. Experimental Planning

### 2.1. Experimental Equipment and Materials

**Figure 1:**
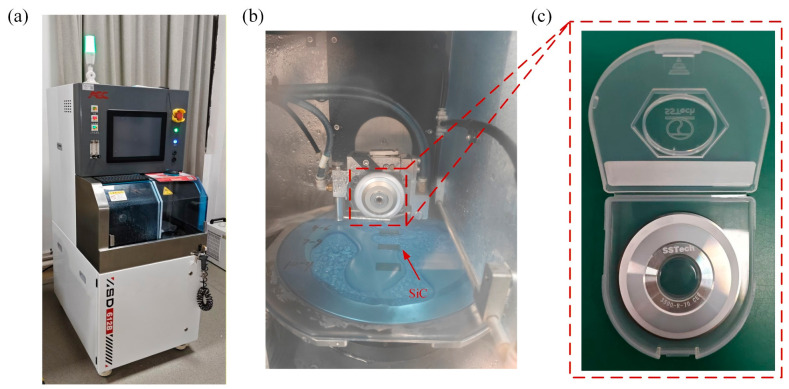
Experimental equipment and blade types: (**a**) precision dicing machine; (**b**) SiC dicing process; (**c**) hard blade.

The experiment utilized an SD612B precision diamond wheel dicing machine, with a high-precision vision positioning system and accurate control capability, as shown in Figure 1a. The X, Y, Z, and θ axes of the dicing machine offer high adaptability with a positioning accuracy of 1–2 μm, ensuring reliable stability during the dicing process. SiC wafers with thicknesses of 540 ± 25 μm served as the experimental material. The wafers were mounted on a workbench using blue adhesive film and secured using a vacuum chuck under a vacuum pressure of 85~90 kPa. The slitting blade employed was a hard-blade-type with an electroplated wheel hub manufactured by SST (Zhejiang Xiste Technology Co., Ltd., Jiaxing, China), as shown in Figure 1b,c. Table 1 shows the relevant parameters of the blade. In the dicing process, the layered dicing method is adopted. The first pass of the ultra-thin diamond grinding wheel blade performs dicing with a shallow depth of dicing. After dicing a certain distance, the blade returns without stopping the machine, and then a second pass along the formed groove completely dices through the workpiece and cuts into the blue tape by 0.05 mm. Blade toughness and strength remained stable and controllable, effectively preventing blade breakage during the dicing process. Physical defects such as edge chipping were clearly detected using the X1200 objective of a Super-Depth-of-Field 3D Digital Microscope (Nanjing Kaishimai Technology Co., Ltd., Nanjing, China).

### 2.2. Experimental Principle

**Figure 2:**
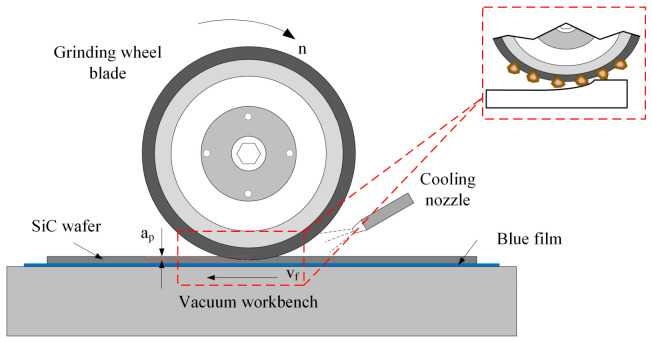
Principle of diamond wheel dicing.

The principle of diamond grinding wheel dicing is shown in Figure 2. The grinding wheel slicing machine operates with a high-speed air-static electric spindle driving blade rotation to dicing the workpiece. Under such conditions, a large number of abrasive grains adhering to the blade surface exert strong mechanical grinding on the SiC surface. Multiple abrasive grains function collectively as a dicing blade to machine SiC, forming a dicing groove along a predetermined path. Deep dicing into the workpiece surface is achieved, thereby removing material. The experiment adopted the down-dicing mode, where the grinding wheel rotation direction aligns with the wafer feed direction. Vacuum negative pressure and water flow remove the chips in a timely manner to prevent scratching the machined surface. High-speed grinding wheel operation generates significant heat, which requires prompt dissipation. In industrial applications, deionized water serves as the cooling medium during the machining process to prevent workpiece damage and deformation. Material removal is accompanied by blade wear. Therefore, blade wear is one of the key indicators of the dicing mechanism [22,23]. After completing the predetermined number of dicing operations, the dicing machine measures the height once and calculates the blade wear amount:

(1) $$∆t=L_{n}-L_{n+1}$$

$L_{n}$: The blade exposure measured at the nth time.
$L_{n+1}$: The blade exposure measured at the (*n* + 1)th height measurement (*n* = 1, 2, 3, …).

During the initial stage of blade wear in the dicing process, the abrasive grains first come into contact with the workpiece, resulting in high wear rates. Harder abrasive grains cause the grinding process to transition to micro-grinding, thereby decreasing the wear of the blade. As abrasive particles wear away and flake off, the binder between them is exposed, which increases the load on the abrasive particles at both ends of the blade, thereby causing the wear rate of the blade to rise gradually. As dicing depth and spindle speed increase, blade wear accelerates due to the increasing frequency of abrasive grain contact. When the dicing process parameters fall within the appropriate range, new abrasive grains are continuously exposed, and blade wear gradually decreases.

**Figure 3:**
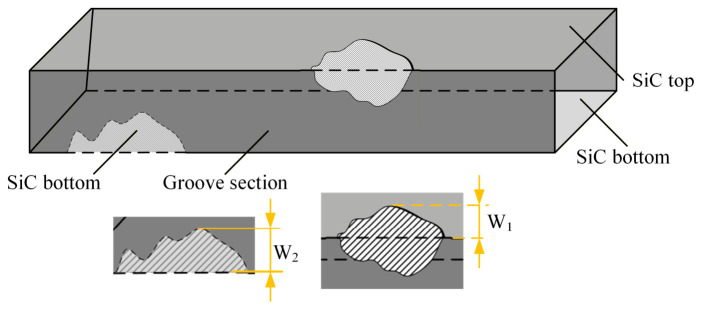
Schematic diagram of measuring quality indicators.

Furthermore, spindle speed, feed rate, and dicing depth exhibit significant interactions that affect dicing quality, making them the focus of the experimental investigation. To investigate the effect of the first dicing depth on workpiece quality, the experiment takes “first dicing depth” as an independent variable. SiC is a typical hard and brittle material that is highly susceptible to chipping along the dicing street during the dicing process, leading to an increase in chipping width. Back-side chipping width is also one of the metrics used to evaluate dicing quality, yet research in this area remains very limited. Therefore, the maximum chip width on the front side W_1_ and the maximum chip width on the back side W_2_ were selected as evaluation criteria. The dicing blade was used to scribe a silicon carbide workpiece along a certain length, forming a scribed groove. A 3D ultra-depth microscope at 1200× magnification was then focused on the top surface of the groove. The width of every single chipping defect along the entire scribe length was measured manually using KS-Studio 3D Ultra-Depth-of-Field Microscope Software (V1.1.2.0). The maximum width among all these measurements was recorded as W_1_. The front surface of the workpiece was attached to blue tape. The blue tape on the back was gently peeled off from the edge. Using the same measurement method, the maximum chipping width on the back surface was measured under a high-magnification 3D ultra-depth microscope and recorded as W_2_, as illustrated in Figure 3.

### 2.3. Basic Experimental Design and Parameter Screening

#### 2.3.1. Basic Experimental Design

**Figure 4:**
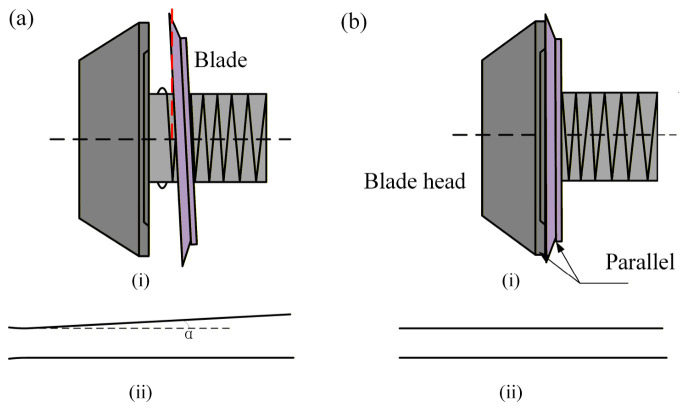
Schematic diagram showing the positional relationship between the blade head face and the blade face: (**a**): (**i**) blades not installed in parallel, (**ii**) dicing path; (**b**): (**i**) blades installed in parallel, (**ii**) dicing path.

Figure 4 illustrates the relative positions of the dicing head face and the dicing blade face. Before dicing the workpiece, the dicing head and dicing blade must be verified for parallelism and proper alignment, and the workpiece must be pre-diced. A certain angle exists between the face of the dicing head and the face of the dicing blade, as shown in Figure 4a. Figure 4b shows the correct installation position of the blade and the blade head. An overall tilt in the dicing path can directly compromise dicing quality. During an emergency blade lift, such tilting may also cause blade chipping, resulting in increased costs. During pre-dicing, blade wear intensifies at a spindle speed of 10,000 rpm and a feed rate of 10 mm/s when the dicing process penetrates the workpiece in a single pass. Relatively slow spindle speed, relatively fast feed rate, and large depth of dicing result in high friction from individual abrasive grains attached to the blade. Such friction increases dicing forces, causing the next abrasive grain to engage before the previous diamond grain has completed its dicing task, thereby expanding the chipped edge. Therefore, within the appropriate range of parameters, a lower feed rate and a higher spindle speed should be selected.

To determine appropriate parameter ranges for spindle speed, feed rate, and the first dicing depth, five spindle speed levels were selected at 16,000, 20,000, 24,000, 28,000, and 32,000 rpm. Five feed rate levels were set at 0.5, 1, 2, 4, and 6 mm/s. Three initial dicing depth levels were established at 170 μm, 270 μm, and 370 μm. In designing the single-factor dicing experiment, the SiC wafer was divided into several chips measuring 10 mm × 20 mm to minimize repeated tape mounting operations and reduce mutual interference between different dicing runs. The dicing length was set to 20 mm with a dicing pitch of 2.5 mm. Each experimental condition was repeated three times, and each dicing operation was measured three times, after which the average value was calculated. By analyzing the effects of the process parameters on the dicing quality indicators, specifically W_1_ and W_2_, the parameter ranges for improved dicing quality were further identified.

#### 2.3.2. Influence Patterns of Single Factors on W_1_ and W_2_ and Parameter Determination

**Figure 5:**
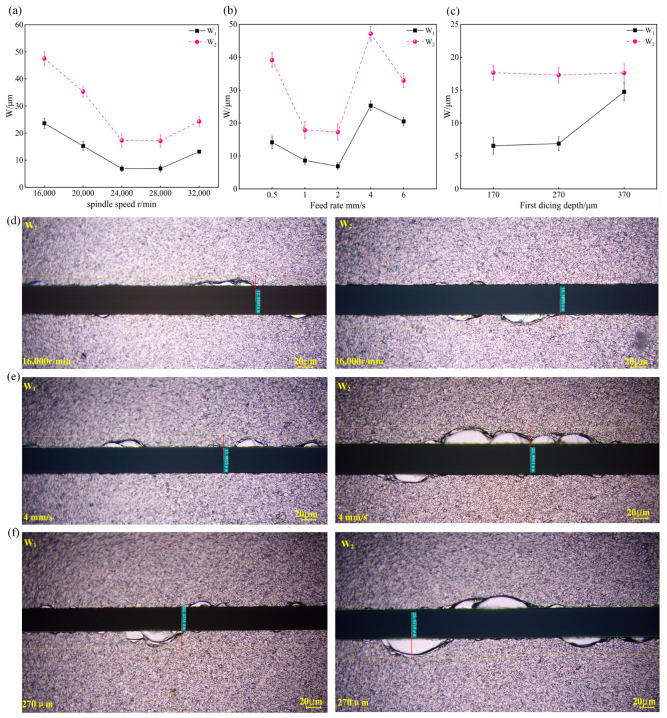
Influence patterns of spindle speed, feed rate, and the first dicing depth with corresponding chipping effects: (**a**–**c**) influence patterns of W_1_ and W_2_; (**d**–**f**) maximum chipping width effects of W_1_ and W_2_.

Figure 5a–f illustrate the effect of spindle speed on the maximum front-side chipping width W_1_ and back-side chipping width W_2_, along with representative chipping width, under varying spindle speeds, feed rates, and the first dicing depths. Among them, Figure 5a shows that both W_1_ and W_2_ first decrease and then increase with increasing spindle speed. Under constant other conditions, when the spindle speed falls within the range of 24,000 to 28,000 r/min, W_1_ and W_2_ remain at low levels and exhibit a gradually decreasing trend. At spindle speeds of 20,000 r/min and 32,000 r/min, W_1_ remains at a high level, while W_1_ and W_2_ gradually increase as the spindle speed approaches 32,000 r/min. To better capture the trend of chipping width variation with spindle speed and align with the preference for higher spindle speeds in dicing operations, the spindle speed was controlled within the range of 24,000 to 32,000 r/min. Figure 5b shows the effect of feed rate on W_1_ and W_2_. With other parameters held constant, W_1_ and W_2_ exhibit similar variation trends as the feed rate increases: first decreasing, then increasing, and finally decreasing again. Both W_1_ and W_2_ remain at low levels at feed rates of 1 mm/s and 2 mm/s, with the near-minimum chipping width occurring at a feed rate of 2 mm/s. Within the feed rate range of 4 mm/s to 6 mm/s, both W_1_ and W_2_ exhibit a rapid, decreasing trend. To balance dicing quality with dicing efficiency, feed rates were selected within the range of 2 mm/s to 6 mm/s. With a shallow dicing depth in the first pass, the force exerted on the dicing blade remains relatively low. During the second pass, however, the greater dicing depth results in a higher dicing force, and greater force is transmitted to the workpiece. These conditions promote crack formation along both sides of the dicing kerf, thereby exacerbating chipping. Figure 5c shows the effect of different first dicing depths on W_1_ and W_2_. At the first dicing depth of 270 μm, W_1_ and W_2_ remain relatively low. Further increases in dicing depth lead to growth in both chipping widths. The first dicing depth clearly exerts a substantial influence on chipping width. To better assess how the interaction between dicing depth and the other two parameters affects W_1_ and W_2_, the first dicing depth was selected within the range of 170 μm to 370 μm.

## 3. Response Surface Experimental Design and Result Analysis

### 3.1. Experimental Protocol

Using a single-factor experiment with dicing, the levels for each dicing parameter were determined, as shown in Table 2.

Response surface methodology (RSM) serves as a method for studying mathematical relationships between influencing factors and response variables, involving fundamental theoretical knowledge from applied mathematics, statistical analysis, and experimental design. RSM was originally proposed by Box and Wilson, who developed and derived its mathematical models, subsequently combining them with statistical analysis to create a method suitable for optimizing process parameters [24]. Therefore, the response surface method was selected to establish the following equation for predicting the response variable:

(2) $$W_{n}=\beta_{0}+\sum_{i=1}^{m}\beta_{i}x_{i}+\sum_{i=1}^{m}\beta_{ii}x_{i}^{2}+\sum_{i=1}^{m}\sum_{i<j}^{m}\beta_{ij}x_{i}x_{j}+\varepsilon_{c}$$

W_n_ represents the maximum front-side chipping width or the maximum back-side chipping width (*n* = 1, 2). The symbols *x_i_* and *x_j_* represent the input process parameters, including spindle speed, feed rate, and the first dicing depth. The variable m denotes the number of input variables and equals 3. The symbols *β*_0_, *β_i_*, *β_j_*, and *β_ij_* represent the coefficients of the constant term, the linear effect terms, the quadratic effect terms, and the interaction effect terms, respectively. The symbol *ε_c_* (*c* = 1, 2) denotes the modeling error. A three-factor, three-level response surface experiment was designed using Design Expert software (Design-Expert 13). The response variables were defined as the maximum front-side chipping width W_1_ and the maximum back-side chipping width W_2_. To ensure the representativeness of each parameter combination, the wafer was diced three times under each experimental condition, and the measured results are summarized in Table 3.

### 3.2. Response Surface Analysis

#### 3.2.1. Model Establishment and Variance Analysis

After approval of the response surface experiment design, the effects of each parameter (W_1_ and W_2_) were analyzed. Table 4 below shows the analysis of variance (ANOVA) for the response volume W_1_ and W_2_ as functions of spindle speed (A), feed rate (B), and the first dicing depth (C).

The reliability of the model was evaluated using analysis of variance (ANOVA), as shown in Table 4. In the ANOVA table, the F-value represents the ratio of between-group variance to within-group variance, thereby quantifying the strength of the relationship between each process parameter and the response variable. A higher F-value indicates a greater overall effect of the corresponding parameter. As a statistical measure, the *p*-value operates such that a smaller value reflects stronger statistical significance and a better model fit. Research indicates that a *p*-value less than or equal to 0.05 denotes statistical significance, and a *p*-value less than or equal to 0.001 denotes high significance.

According to Table 4, the W_1_ regression model yields a *p*-value of 0.0031, while that for W_2_ is 0.0012, confirming the statistical significance of both quadratic regression models. The lack-of-fit *p*-values for W_1_ and W_2_ are 0.8812 and 0.6777, respectively. Such high values indicate no significant lack of fit, meaning the discrepancy between the fitted equations and the actual data remains minor. Consequently, both regression models are considered valid. Regarding the significance of individual factors and interaction terms for W_1_, most terms exert a notable influence, with the exception of BC and B^2^. Given that the *p*-values for BC and B^2^ exceed 0.1, variations in these two terms contribute little to overall changes in W_1_. Nevertheless, BC and A^2^ are retained in the model equation, as shown in Equation (3), to preserve model integrity. For the quadratic regression model of W_1_, the unadjusted R^2^ stands at 0.9683, and the adjusted R^2^ at 0.9111. The small gap between these two values indicates a strong model fit. Moreover, the predicted R^2^ of 0.8231 deviates from the adjusted R^2^ by less than 0.2, suggesting that the model not only has strong predictive power but also maintains reasonable internal consistency.

Significance analysis of individual factors and interactions on W_2_ was also conducted. As shown in the analysis of variance presented in Table 4, all factors except A^2^ and C^2^ are found to have a significant effect on W_2_. Given that *p*-values for both A^2^ and C^2^ exceed 0.1, variations in these two terms contribute little to overall changes in W_2_. Nevertheless, A^2^ and C^2^ remain in the model equation, as shown in Equation (4), to preserve model integrity. For the quadratic regression model of W_2_, unadjusted R^2^ equals 0.9781, while adjusted R^2^ equals 0.9387. A small gap between these two values indicates a strong model fit. Furthermore, the predicted R^2^ value of 0.8093 differs from the adjusted R^2^ by less than 0.2, suggesting good consistency and reliable predictive capability without signs of overfitting.

(3) $$W_{1}=288.40155-0.013715\times A-3.57520\times B-488.07159\times C+0.000239\times A\times B+0.012456\times A\times C\;-6.00504\times B\times C+1.53782\times 10^{-7}\times A^{2}-0.077836\times B^{2}+251.04554\times {C\;}^{2}$$

(4) $$W_{2}=321.10518-0.012105\times A-26.90055\times B-373.56145\times C+0.000907\times A\times B+0.013294\times A\times C\;-18.75962\times B\times C+7.33188\times 10^{-8}\times A^{2}+0.674225\times B^{2}+6.79250\times C^{2}$$

Note: The unit of C is mm.

#### 3.2.2. Response Surface Analysis of Various Factors on W_1_

**Figure 6:**
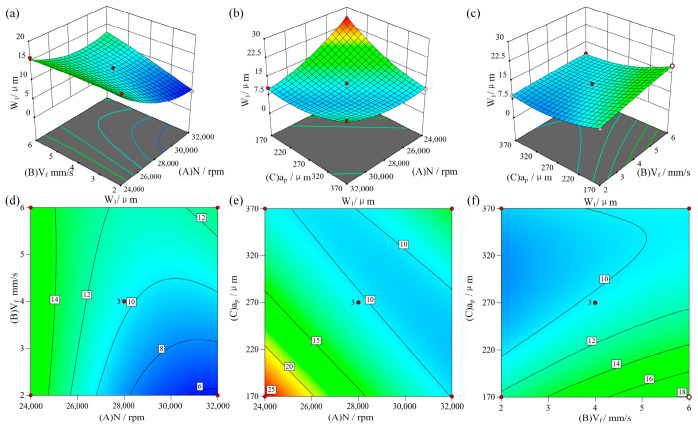
Response surface and contour plot of process parameters on W_1_: (**a**) Response surface model of N-V_f_ interaction; (**b**) Response surface model of N-a_p_ interaction; (**c**) Response surface model of V_f_-ap interaction; (**d**) Contour plot of N-V_f_ interaction; (**e**) Contour plot of N-a_p_ interaction; (**f**) Contour plot of V_f_-a_p_ interaction. Note: The colors indicate the magnitude of the predicted response, with darker or redder colors corresponding to larger predicted values. The numbers on the contour plots denote the response contour levels.

Figure 6 presents response surface curves and contour plots for W_1_ under varying process parameters. According to the ANOVA table, the AB interaction term yields a *p*-value of 0.0481, confirming that the interaction between spindle speed and feed rate exerts a significant influence on W_1_. With the first dicing depth held constant, W_1_ rises with an increasing feed rate while gradually declining as spindle speed increases. However, as shown in Figure 6a, variation in feed rate produces a maximum difference of roughly 0.5 μm across the two ends of W_1_, following a nearly linear trend. Such behavior indicates that changes in feed rate exert a relatively weak influence on W_1_. In contrast, variation in spindle speed displays a steep trend relative to W_1_, revealing a strong overall correlation. Figure 6d reveals that, with increasing spindle speed, the contour line gradient for W_1_ becomes steeper in the direction of decreasing feed rate, while W_1_ shows a decreasing trend, indicating better performance. Such behavior arises mainly because feed rate alone exerts a weak correlation with W_1_; spindle speed plays a dominant role during the dicing of silicon carbide. A low feed rate causes the abrasive grain to repeatedly scrape a small area to remove a large material volume, given the short contact time between the grain and the workpiece and the small work volume. Such an action leads to grain wear and a reduction in undeformed chip thickness, thereby lowering normal and tangential forces acting on each abrasive grain. As a result, the widening effect on the dicing groove of silicon carbide diminishes, producing a decrease in W_1_. According to the contour plot in Figure 6d, improved W_1_ performance occurs within a feed rate range of 2~3 mm/s and a spindle speed range of 28,000~32,000 r/min.

Figure 6b,e reveal ridge-like response surfaces for spindle speed and dicing depth, indicating a substantial interaction between these two parameters. At a feed rate of 4 mm/s, changes in spindle speed and first dicing depth show a strong correlation with W_1_. Increasing both parameters produces a high–low–high distribution of W_1_ across both ends. Figure 6e clearly shows that, with a spindle speed near 32,000 r/min, a smaller difference in the first dicing depth between two passes leads to better control of chipping width W_1_ on both sides of the dicing path. This trend follows the secondary diagonal direction of dicing depth contour lines. Such behavior occurs because, at high speed and with a small difference in dicing depth between two passes while maintaining dicing efficiency, each abrasive grain cuts at a consistent depth. Consequently, the dicing edge experiences uniform compressive forces from both sides during the dicing process, resulting in lower internal stress within the workpiece. Figure 6c,f show generally flat response surfaces without obvious peaks or troughs. Additionally, *p*-values from the ANOVA table exceed 0.05, confirming a lack of significant effect on maximum dicing-induced crack width W_1_.

#### 3.2.3. Response Surface Analysis of Various Factors on W_2_

**Figure 7:**
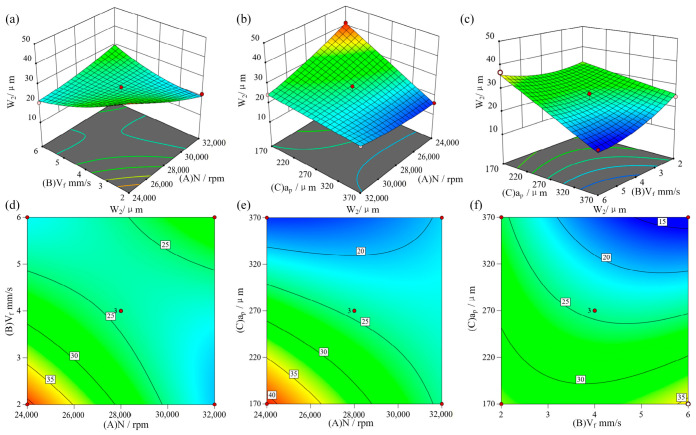
Response surface and contour plot of process parameters on W_2_: (**a**) Response surface model of N-V_f_ interaction; (**b**) Response surface model of N-a_p_ interaction; (**c**) Response surface model of V_f_-ap interaction; (**d**) Contour plot of N-V_f_ interaction; (**e**) Contour plot of N-a_p_ interaction; (**f**) Contour plot of V_f_-a_p_ interaction. Note: The colors indicate the magnitude of the predicted response, with darker or redder colors corresponding to larger predicted values. The numbers on the contour plots denote the response contour levels.

Figure 7 presents response surface and contour plots for W_2_ under varying dicing process parameters. Figure 7a illustrates the interaction curve between spindle speed and feed rate in relation to W_2_. Along the diagonal of the response surface within the high-spindle-speed and high-feed-rate region, the variation in dicing chip width W_2_ follows a peak-shaped pattern with low–medium–low variation, and the optimal chip width occurs at both endpoints. Regardless of parameter selection, W_2_ remains relatively large. Insufficient heat dissipation during the dicing process primarily causes this behavior, as chips adhere to the end faces of the dicing path and produce secondary dicing. Beyond material removal by abrasive grains on the dicing edge, chips that remain lodged also exert pressure on back-face cracks. Upon contact of the dicing edge with the back side of the workpiece, the material on either side of the dicing path exerts opposing tensile and compressive forces as the blade transitions from the workpiece to the film, thereby inducing brittle fractures. When transverse cracks created by abrasive grains within the workpiece reach a significant size, resulting damage to the workpiece becomes difficult to control, and the corresponding W_2_ also remains relatively large. Figure 7d reveals the maximum W_2_ value under conditions where both the spindle speed and feed rate reach minimum levels. Within a given unit of time, overlap between low material removal rates and extremely high abrasive action causes the dicing process to shift from effective dicing to harmful extrusion and plowing, leading to the accumulation of fatigue damage within the material.

The degree of contour curvature in the response surface reflects the correlation strength between two factors. Figure 7b demonstrates that the first dicing depth exerts a more significant influence on W_2_ than the spindle speed. At a constant feed rate, W_2_ decreases with increasing spindle speed and first dicing depth. The distribution of dicing depth affects the amount of material removed by the dicing edge during the initial dicing pass. When the first dicing depth is large, significant heat generated from a deep dicing pass may cause insufficient chip evacuation, leading to chip accumulation. During the second dicing pass, however, the depth of dicing per abrasive grain remains shallow, reducing the impact on the workpiece. Accumulated chips are also cleared away. Reducing stress on the blade’s back face consequently yields a relatively narrower chipping width. When the first dicing depth remains small, the secondary dicing depth becomes large, the spindle speed remains low, and the force exerted by abrasive grains striking the workpiece increases, thereby promoting the propagation of transverse cracks at the bottom of the silicon carbide. At the moment that the dicing edge removes material, the constraint on the bottom edge reduces, allowing cracked material to break off, and W_2_ reaches its maximum value. Figure 7e, a contour plot, shows that at a feed rate of 4 mm per second, the dicing depth remains in a range between 320 and 370 μm, and the value of W_2_ is reasonable. Figure 7c reveals that when the spindle speed is fixed at 28,000 r/min and within the selected parameter range, W_2_ decreases as the first dicing depth and feed rate increase, while the first dicing depth exerts a more significant influence on W_2_. This trend arises primarily because an increased dicing depth and feed rate produce a higher material removal rate, shortening the sliding time of abrasive grains across the silicon carbide workpiece’s surface and reducing repeated plowing and squeezing events on the same area, thereby limiting the accumulation of subsurface fatigue damage. At a low dicing depth and low feed rate, abrasive grains repeatedly act on the same area of the silicon carbide dicing surface, causing the accumulation of damage layers. Such conditions readily lead to large-scale spalling at the back-side exit. Figure 7f reveals the optimal W_2_ when the feed rate ranges from 3 to 6 mm/s and the dicing depth ranges from 320 to 370 μm.

### 3.3. Process Parameter Optimization and Experimental Verification

**Figure 8:**
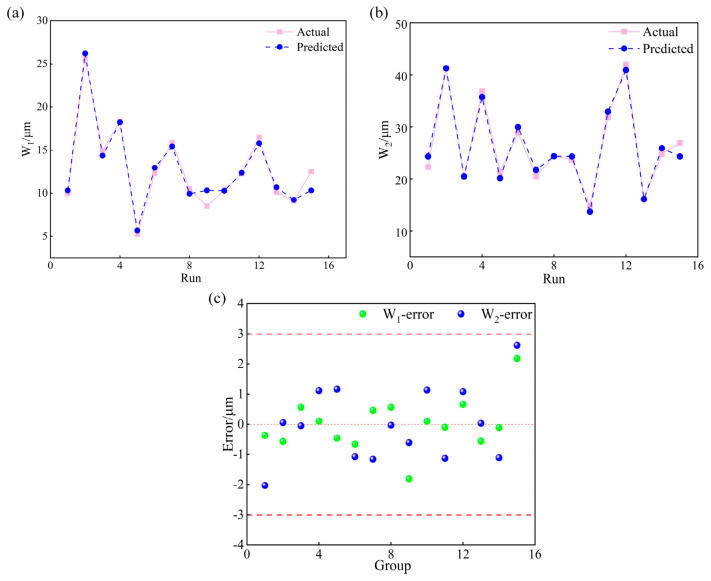
Model fitting error plots: (**a**) actual vs. predicted W_1_; (**b**) actual vs. predicted W_2_; (**c**) error ranges for W_1_ and W_2_. Note: The red dashed lines in Figure (**c**) represent only the upper and lower error limit values.

Figure 8a,b demonstrate the fit between the quadratic regression prediction model and actual measured data. The predicted data line generally aligns well with the actual data line, confirming the effective predictive performance of the model for W_1_ and W_2_. Figure 8c presents a scatter plot of error ranges between the predicted and actual values for W_1_ and W_2_. The overall error remains within ±3 μm, showing a relatively uniform distribution pattern. These results demonstrate the accuracy and reliability of the model in deciphering the experimental data.

Multi-objective optimization primarily involves maximizing or minimizing different objective functions under a set of constraints. Such an optimization problem yields a set of solutions. For a set of solutions to multiple objective functions, direct comparison of relative merits between solutions proves impossible. These solutions are known as non-dominated solutions or Pareto solutions. Typically, practical analysis determines the priority of these objectives. The NSGA-II multi-objective genetic optimization algorithm improves upon the non-dominated sorting genetic algorithm (NSGA) through the introduction of an elite retention strategy, effectively accelerating convergence to the Pareto-optimal frontier while better preserving genetic diversity [25]. This experiment adopted the maximum front chipping width W_1_ and the maximum back chipping width W_2_ as minimization objectives. The NSGA-II multi-objective genetic optimization algorithm was utilized with a population size set to 100, an iteration number of 500, a crossover rate of 0.7, and a mutation rate of 0.01. The optimization objectives and constraint conditions for the response variables are as follows:

$$\min\{W_{1},W_{2}\}\;\;s.t.\left\{\begin{array}{c} 24,000\leq x_{1}\leq 32,000;\;rpm\\ 2\leq x_{2}\leq 6;\;mm/s\\ 170\leq x_{3}\leq 370;\;\mu m \end{array}\right.$$

**Figure 9:**
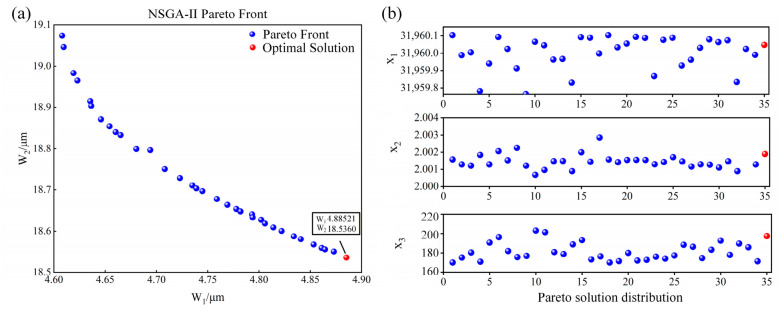
NSGA-II algorithm optimization: (**a**) Pareto-feasible solutions and optimal solution; (**b**) distribution of x_1_, x_2_, x_3_.

The following results were obtained after NSGA-II multi-objective genetic algorithm optimization:

Figure 9a shows a generally even distribution of feasible solutions for response variables A, B, and C, along with optimization objectives W_1_ and W_2_, after NSGA-II genetic algorithm optimization. As W_1_ increases, W_2_ displays a gradual decreasing trend. Within the feasible region, W_1_ ranges from 4.60 to 4.90 μm, and W_2_ ranges from 18.5 to 19.1 μm. No Pareto-feasible solution exists when both objective functions reach minimum values simultaneously in Figure 9b. Among the Pareto solutions with respect to the coordinate origin (W_1_ = 4.60 μm, W_2_ = 18.5 μm), the left boundary value in Figure 9a gives the minimum W_1_ while producing a comparatively large W_2_, with a deviation of 0.57 μm from the lowest W_2_ value. Taking an intermediate local value results in neither a minimum W_1_ nor a minimum W_2_. Taking the right boundary value yields the minimum W_2_ while causing only a relatively small change of 0.28 μm in W_1_. In engineering dicing, the chipping width W_2_ presents a control difficulty. To minimize W_2_ while maintaining a relatively small W_1_ and considering surface quality, an appropriate parameter set consists of a spindle speed of 31,960 r/min, a feed rate of 2.0019 mm/s, and a depth of dicing of 197.51 μm. Owing to the precision limitations of the equipment spindle, the following spindle speeds were rounded to 31,960 r/min in the experiments.

**Figure 10:**
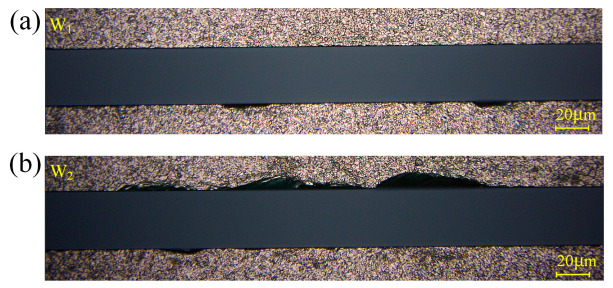
Experimental verification of dicing street morphology: (**a**) W_1_; (**b**) W_2_.

Based on the optimized experimental data described above, dicing tests were conducted for result verification. To minimize random experimental errors, experimental group data came from three separate dicing tests and underwent comparative analysis. Figure 10 shows the groove morphologies of the front and back surfaces of the workpiece after dicing. The positive dicing path shows no large chipping notches, indicating good performance. Maximum chipping width decreased by 80.38% compared to the pre-optimization value. However, the maximum back chipping width still exhibits large chipping defects, deep chipping, and complete edge chipping. The primary cause lies in the large dicing force exerted on the blade throughout the dicing process. At the moment of dicing through the workpiece, the dicing edge experiences uneven force distribution and potential deflection, which leaves the maximum back chipping width moderately large. Nonetheless, compared to the pre-optimization value, the overall maximum back chipping width decreases by 53.81%, indicating convergence and a favorable optimization outcome. Table 5 compares the predicted values and the average experimental values of W_1_ and W_2_ obtained from validation experiments following NSGA-II genetic algorithm optimization. The resultant relative errors are 2.83% for W_1_ and 4.43% for W_2_, both below 5%, thereby confirming the accuracy of the prediction model.

## 4. Conclusions

This experimental study employed response surface analysis to investigate how process parameters affect the maximum front chipping width W_1_ and the maximum back chipping width W_2_ during the precision diamond blade dicing of silicon carbide. The main conclusions are as follows:

(1) Spindle speed ranks as the most critical factor affecting maximum chip width W_1_, followed by the first dicing depth, with feed rate being the least influential. Based on the single-factor experiment, W_1_ increases with increasing spindle speed and increasing first dicing depth, although the magnitude of change remains relatively small. Within a certain range, the interaction of feed rate with spindle speed and first dicing depth can lead to an overall improvement in W_1_. When the spindle speed was 28,000 rpm, the feed rate was 2 mm/s, and the first dicing depth was 270 μm, W_1_ measured less than 9.14 μm.
(2) In the layered dicing process, the difference in the first dicing depth directly affects the extent of variation in W_1_ and W_2_. Under a spindle speed of 24,000 r/min and a feed rate of 2 mm/s, the front maximum chipping width W_1_ initially decreases and then increases with the first dicing depth, while the back maximum chipping width W_2_ first increases slowly and then rapidly as the first dicing depth increases.
(3) Preventing the combination of low spindle speed and low feed rate can effectively prevent W_2_ from becoming excessively large. By strictly controlling the first dicing depth, the accumulation of subsurface damage inside the workpiece can be reduced, thereby suppressing the expansion of the back-side chipping width. In the response surface model, when the spindle speed was held constant, the back maximum chipping width W_2_ decreased rapidly with simultaneous increases in the first dicing depth and the feed rate, reaching a minimum of 14.83 μm.
(4) Based on the response surface analysis, quadratic regression models for W_1_ and W_2_ were established. The NSGA-II multi-objective optimization algorithm was employed to determine the optimal parameter combination, which was then validated experimentally. The resulting relative errors for W_1_ and W_2_ were 2.83% and 4.43%, respectively, both falling within 5%. These results confirm the validity of the prediction models for W_1_ and W_2_ and provide a reliable reference for improving the dicing quality of silicon carbide in the future.

## Figures and Tables

**Table 1:** The specific parameters of the dicing blade.

Hard Blade Series	SSTYE	Concentration	70
Abrasive type	SD Synthetic Diamond	Blade Length/mm	0.89~1.02
Abrasive Grain Size	3500	Kerf Width/mm	0.040~0.050
Bond Hardness	R Hard	Blue Film SPV 224SRB	t = 0.08 mm

**Table 2:** Process parameters.

Factor Name	Low Level	Medium Level	High Level
Spindle Speed r/min	24,000	28,000	32,000
Feed Rate mm/s	2	4	6
Dicing Depth/μm	170	270	370

**Table 3:** Experimental scheme and test data.

Index	Response Variable			Response Volume	
	*N* r/min	*V_f_* mm/s	*a_p_*/mm	*W*_1_/μm	*W*_2_/μm
1	24,000	2	0.27	16.4600	41.9884
2	32,000	2	0.27	5.2321	21.3061
3	24,000	6	0.27	15.8926	20.5339
4	32,000	6	0.27	12.3021	28.8760
5	24,000	4	0.17	25.6322	41.3085
6	32,000	4	0.17	10.5029	24.3427
7	24,000	4	0.37	10.1196	16.1161
8	32,000	4	0.37	14.9193	20.4212
9	28,000	2	0.17	12.2723	31.8096
10	28,000	6	0.17	18.3310	36.8518
11	28,000	2	0.37	9.1143	24.7939
12	28,000	6	0.37	10.3690	14.8284
13	28,000	4	0.27	9.9527	22.2849
14	28,000	4	0.27	8.5128	23.7037
15	28,000	4	0.27	12.5022	26.9297

Note: *N* represents the spindle speed, *V_f_* represents the feed rate, and *a_p_* represents the first dicing depth. In the table, the unit of *a_p_* is given in mm, whereas in the rest of the text, all values of the depth of dicing are expressed in μm.

**Table 4:** Variance analysis table for W_1_ and W_2_ quadratic regression models.

Source	W_1_		W_2_	
	F	*p*	F	*p*
Model	16.94	0.0031	24.83	0.0012
A (Spindle speed)	36.72	0.0018	18.27	0.0079
B (Feed rate)	11.08	0.0208	10.34	0.0236
C (First dicing depth)	28.66	0.0031	98.84	0.0002
AB	6.77	0.0481	49.25	0.0009
AC	46.12	0.0011	26.45	0.0036
BC	2.68	0.1625	13.17	0.0151
A^2^	10.38	0.0234	1.19	0.3254
B^2^	0.1663	0.7003	6.28	0.0541
C^2^	10.81	0.0218	0.004	0.9521
Lack of Fit	0.2124	0.8812	0.5914	0.6777
R^2^	0.9683	/	R^2^	0.9781
Adjusted R^2^	0.9111	/	Adjusted R^2^	0.9387
Predicted R^2^	0.8231	/	Predicted R^2^	0.8093

**Table 5:** Test verification data.

Experimental Group	Group 1	Group 2	Group 3	Average Value	Predicted Value	|Absolute Error|	Relative Error
W_1_/μm	5.2043	5.0142	4.8637	5.0274	4.8852	0.1422	2.83%
W_1_/μm	18.5033	19.4528	20.2268	19.3943	18.5360	0.8583	4.43%
Process Parameters	Spindle speed: 31,960 r/min; Feed rate: 2.0019 mm/s; First dicing depth: 197.51 μm						

## Data Availability

The original data supporting this study are included in the article.
